# Cerebrospinal fluid monocyte chemoattractant protein 1 correlates with progression of Parkinson’s disease

**DOI:** 10.1038/s41531-020-00124-z

**Published:** 2020-09-03

**Authors:** Anna Santaella, H. Bea Kuiperij, Anouke van Rumund, Rianne A. J. Esselink, Alain J. van Gool, Bastiaan R. Bloem, Marcel M. Verbeek

**Affiliations:** 1 Departments of Neurology Laboratory Medicine, Radboud University Medical Center, and Donders Institute for Brain, Cognition and Behavior, Nijmegen, The Netherlands; 2 Laboratory Medicine, Radboud University Medical Center, and Donders Institute for Brain, Cognition and Behavior, Nijmegen, The Netherlands; 3 Center of Expertise for Parkinson & Movement Disorders, Nijmegen, Radboud University Medical Center, and Donders Institute for Brain, Cognition and Behavior, Nijmegen, The Netherlands

**Keywords:** Parkinson's disease, Prognostic markers

## Abstract

Parkinson’s disease (PD) and multiple system atrophy (MSA) have overlapping symptoms, challenging a correct early diagnosis. Prognostic information is needed to predict disease progression and provide appropriate counseling. Neuroinflammation plays a role in the pathology of both disorders, as shown in genetic and postmortem tissue studies. Monocyte chemoattractant protein 1 (MCP-1) and neuroleukin (NLK) are two inflammatory proteins with potential to serve as biomarkers of the neuroinflammatory process. Here, we aimed to study the biomarker potential of both MCP-1 and NLK protein levels in cerebrospinal fluid (CSF) from a longitudinal cohort study (Radboudumc, Nijmegen, The Netherlands), consisting of PD patients (*n* = 46), MSA patients (*n* = 17) and control subjects (*n* = 52) using ELISA. We also correlated MCP-1 and NLK levels in CSF to several parameters of disease. We showed that MCP-1 levels in CSF positively correlate with PD progression (*ρ* = 0.363; *p* = 0.017) but could not differentiate between PD, MSA, and controls. NLK levels in CSF neither differentiated between PD, MSA, and controls, nor correlated with disease progression. Our results indicate that MCP-1 levels in CSF cannot distinguish between PD, MSA, and controls but correlate with disease progression in PD patients, suggesting that neuroinflammation is associated with clinical progression in PD. The correlation with disease progression was only moderate, so MCP-1 levels in CSF should be included in a larger battery of prognostic biomarkers that also tackle different pathophysiological processes.

## Introduction

Parkinson’s disease (PD) is the second most common neurodegenerative disorder affecting 1% of the worldwide population older than 65 years, and is expected to affect 12 million people by 2040^1–4^. The exact etiology remains unknown but likely involves both genetic and environmental factors. Classically, neural degeneration is associated with presence of Lewy Bodies, which contain aggregates of the protein α-synuclein (α-syn). However, the pathology of the disease also affects numerous fundamental cellular processes such as neuroinflammation, mitochondrial function, protein trafficking and the proteasome-mediated protein degradation^5^. The characteristic PD motor symptoms appear when 50–80% of dopaminergic neurons have already died. These motor symptoms include bradykinesia, resting tremor, and rigidity^6,7^. Establishing a correct diagnosis of PD in its early stages can be challenging since PD shares many clinical features with multiple system atrophy (MSA). MSA is a sporadic, rare, and aggressive neurodegenerative disease with an average clinical course of 9 years from symptom onset to death^8,9^. Pathologically, MSA is associated with cytoplasmic inclusions of abnormally folded α-syn, predominantly in oligodendrocytes. In the parkinsonian form of MSA (MSA-P), neuronal loss mainly occurs in the nigrostriatal pathway^8,9^. Clinically, MSA-P is an akinetic-rigid syndrome characterized by autonomic dysfunction, gait disturbance, rigidity, progressive bradykinesia, and a poor response to dopaminergic therapy^10^.

To determine treatment strategies and to counsel patients appropriately, an early and reliable differential diagnosis of PD versus MSA is needed. Ideally, clinicians should also have tools to predict disease progression in these patients^11,12^. Currently, the diagnosis is based on clinical examinations made by movement-disorder specialists, who still reach error rates as high as 24%^10^. Since the gold standard diagnosis is postmortem brain tissue examination, there is a need to discover good biomarkers for diagnosis at early disease stages, and to establish biomarkers that may predict disease progression.

Aggregates of α-syn activate microglia and astrocytes, inducing an immune response. Chronic activation of microglia and astrocytes causes a constant release of pro-inflammatory proteins that enhance neuroinflammation and neurotoxicity, leading to cell death^5,13^. Thus, we hypothesized that inflammatory proteins present in the cerebrospinal fluid (CSF) may reflect this chronic state of neuroinflammation, and might be good candidate biomarkers to differentiate PD from MSA, and to predict disease progression. Monocyte chemoattractant protein 1 (MCP-1) is a cytokine that recruits monocytes and T cells to the sites of inflammation. Here, we specifically further investigate its diagnostic and prognostic value in PD and MSA. MCP-1 levels in blood are elevated in PD compared to controls and correlate with PD progression^14^. In other studies, upregulated MCP-1 expression in brain tissue and higher levels in CSF from Alzheimer’s disease patients compared to controls were reported, and this upregulation correlated with disease progression^15,16^. Neuroleukin (NLK) is a neurotrophic factor with axonal growth activity and a product of lectin-stimulated T cells^17,18^. Previous studies showed that NLK is upregulated in the brain in patients with Huntington’s disease^19^ and promotes axonal growth in spinal cord injury^20^, indicating a role of NLK in neurodegenerative processes. Therefore, in the present study, we aimed to assess the potential of the inflammatory proteins MCP-1 and NLK in CSF as biomarkers for diagnosis and progression of PD and MSA.

## Results

**Fig. 1:**
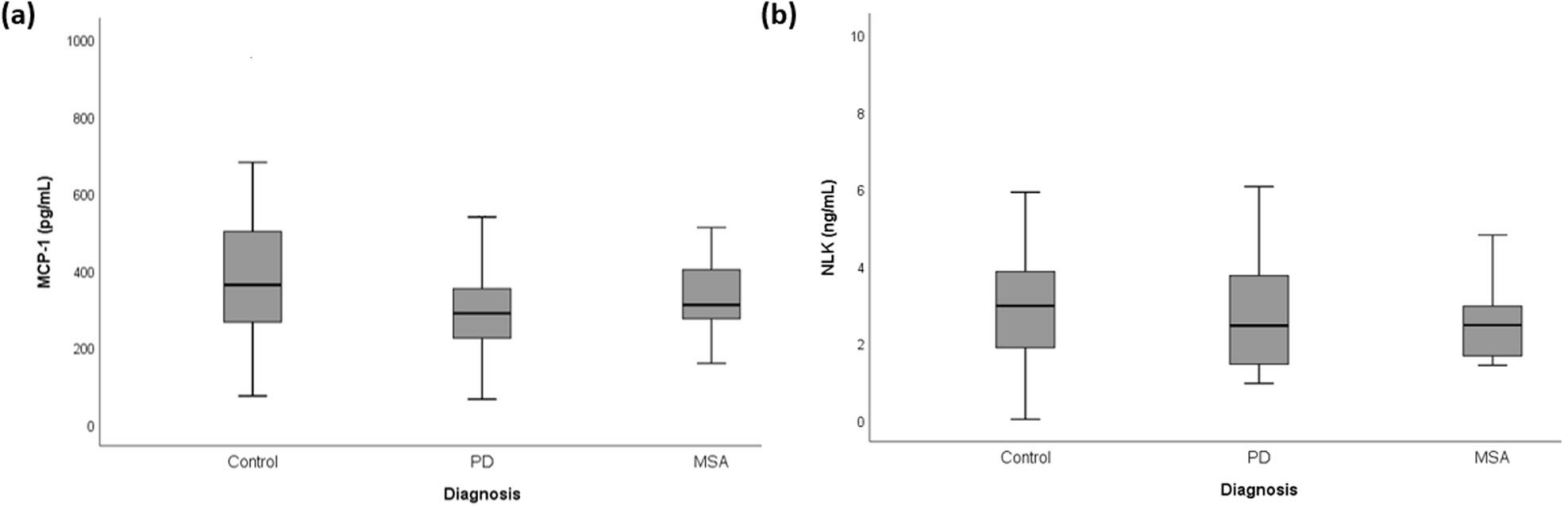
Monocyte chemotactic protein-1 (MCP-1) and Neuroleukin (NLK) levels in CSF. No differences in **a** MCP-1 and **b** NLK levels in CSF were observed between controls, patients with Parkinson’s disease (PD) and patients with multiple system atrophy (MSA). Data were analyzed using rank analysis with age as covariant followed by ANOVA with Hochberg as a post hoc test. Boxplot plots represent median and interquartile range.

We analyzed the levels of MCP-1 and NLK in CSF from 46 PD, 17 MSA, and 52 controls. We first compared the MCP-1 and NLK levels in CSF between clinical groups. The levels of both MCP-1 and NLK did not differ between groups (Fig. 1).

**Fig. 2:**
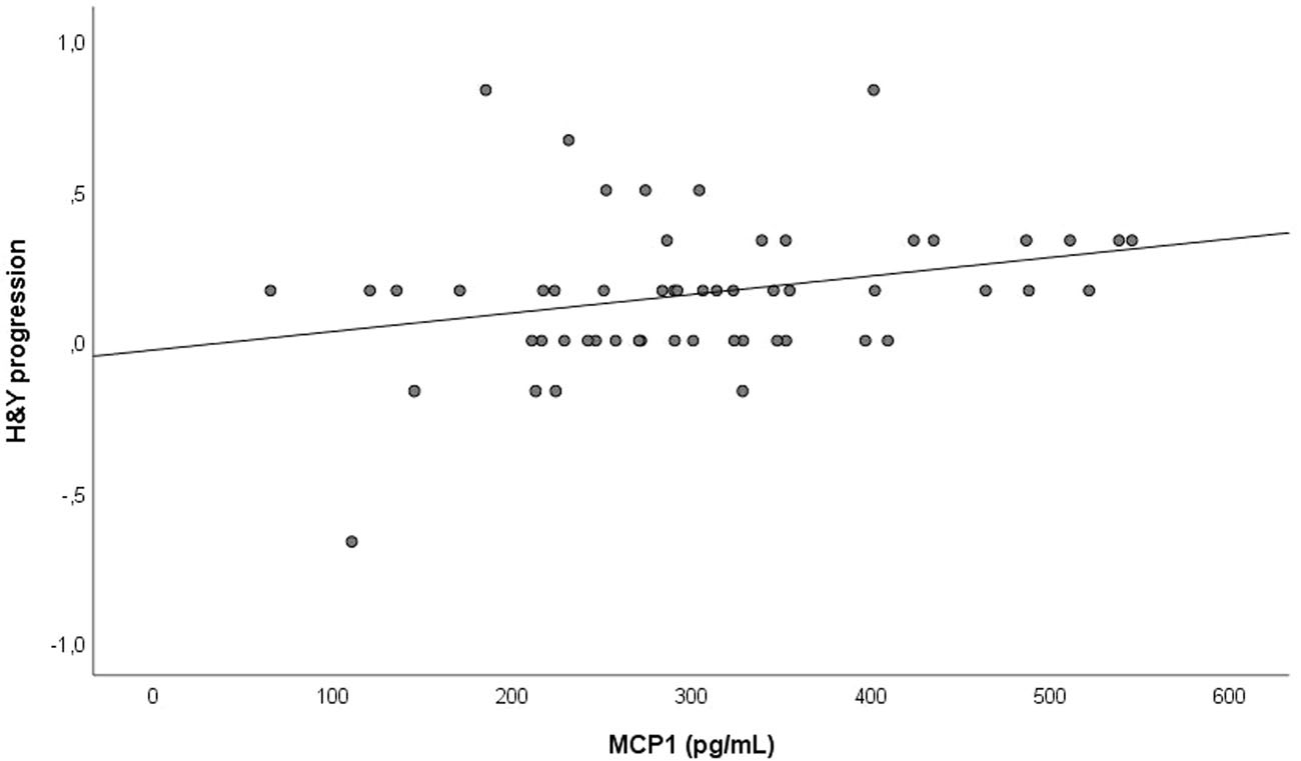
Correlation of monocyte chemoattractant protein-1 (MCP-1) with disease progression. MCP-1 CSF levels at baseline positively correlated with Hoehn and Yahr (HY) progression in the Parkinson’s disease (PD) group. Data were analyzed using Spearman correlation. *ρ* = 0.363 and *p* value = 0.017.

We then correlated MCP-1 and NLK levels in CSF with parameters of disease progression. MCP-1 levels in CSF positively correlated with the change in HY score over a period of 3 years in the PD group (*ρ* = 0.363, *p* value = 0.017, *n* = 43) (Fig. 2). However, this correlation was lost at 10-year follow-up (*ρ* = 0.043, *p* = 0.838, *n* = 25), probably because of the small number of patients. We neither observed correlations between MCP-1 and UPDRS, ICARS or MMSE progression over 3 years in the PD group nor with any of the scales in the MSA group. We did not observe a correlation between NLK CSF levels and any of the disease progression parameters for either PD or MSA (data not shown). We also correlated MCP-1 and NLK levels in CSF with parameters of disease severity at baseline and 3-year follow-up. MCP-1 levels in CSF positively correlated with HY score at 3-year follow-up (*ρ* = 0.459, *p* = 0.002) in the PD group. NLK levels in CSF positively correlated with baseline UPDRS-III (*ρ* = 0.536; *p* = 0.027) and baseline ICARS (*ρ* = 0.596; *p* = 0.032) in the MSA group.

## Discussion

Several studies showed that the levels of the inflammatory proteins MCP-1 and NLK are altered in neurodegenerative diseases, such as PD and AD^14–16,21^ for MCP-1, and multiple sclerosis (MS) and HD for NLK^20,22^.

MCP-1 is a chemokine expressed by glial cells and neurons, and involved in the recruitment of astrocytes, microglia and infiltrating cells from the blood. The MCP-1 receptor (CCR2) is constitutively expressed in brain areas associated with dopaminergic and cholinergic neurotransmission, which are the most affected in PD. Preclinical studies in mouse models suggest that MCP-1 causes neuronal loss and that its downregulation is neuroprotective^23,24^. Thus, we anticipated that CSF levels of MCP-1 would be increased in PD and/or MSA compared to controls. However, and in agreement with other studies^25–29^, we found that CSF levels of MCP-1 are comparable in PD, MSA and controls. In contrast, other studies described increased levels of MCP-1 in CSF of either PD or MSA patients compared to controls^30,31^. An explanation for these discrepancies might be differences in clinical features and pathological state across the various cohorts of patients. In addition, in our study, CSF was collected when there was uncertainty of diagnosis, and diagnosis was reviewed after an extended follow-up period of time. In contrast, other studies analyzed CSF of patients with more advanced disease and shorter follow-up. Finally, different assay types were used to quantify MCP-1 levels in the various studies. One study found an association between MCP-1 and more severe non-motor symptoms in PD^26^. Also, higher MCP-1 expression has been demonstrated in patients with dementia^32,33^. Thus, MCP-1 levels might be higher in later stages of the disease and be associated with disease progression, as we also observed previously^29^. In the present study, we found a weak positive correlation in the PD group between MCP-1 levels in CSF and both final HY score at 3-year follow-up and progression of this score over a 3-year period. This may indicate that a more advanced degree of neuroinflammation may be associated with more advanced motor dysfunction in PD. However, the weak correlation of MCP-1 levels with the HY scores was not supported by UPDRS-III score suggesting that MCP-1 has limited predictive power on its own, but may help as part of a bigger panel that includes a broader range of molecular, imaging, or clinical parameters that also tackle different pathophysiological processes. Unfortunately, the correlation between MCP-1 and HY progression in the PD group was lost after 10-year follow-up. This result might be biased by the lower number of clinical data after 10 years. Alternatively, it might indicate that long-term disease progression may be affected by additional factors.

We did not observe a difference in UPDRS-III progression between PD and MSA patients, and, moreover, we observed that the UPDRS-III progression in patients with MSA was remarkably low. This could be explained by the following observations: (1) 10 of the initial 17 MSA patients could not complete the UPDRS-III assessment at 3-year follow-up (deceased or too severely disabled); (2) 2 of them had more prominent cerebellar features and less pronounced parkinsonian features; and (3) 2 of the 7 patients with follow-up were scored ‘on’ dopaminergic medication because skipping doses of dopaminergic medication would lead to unacceptable deterioration in these patients. However, further analysis on the progression of the complete cohort of PD and parkinsonism patients at 3- and 12-year follow-up showed that the degree of UPDRS-III progression in our cohort is similar as in other longitudinal studies. Noteworthy, recent observations in the large Parkinson’s Progression Markers Initiative cohort have shown a substantial amount of error variance and individual fluctuations of the UPDRS score and therefore, UPDRS might not be precise enough to assess disease progression^34–36^.

NLK is neurotrophic factor with axonal growth activity^17,18^. When located intracellularly, NLK is involved in the insulin-like growth factor 1 (IGF-1)/insulin-like signaling pathway^18,37^, which has been implicated in neurodegeneration and human aging. NLK may suppress α-syn accumulation and toxicity, dopamine neurodegeneration and inflammation in *Caenorhabditis elegans* and *Drosophila*^37^. These studies suggest protective effects NLK in neurodegenerative diseases. Despite the possible association of NLK with the pathology of α-synucleinopathies, our data suggest that NLK neither has a role as a biomarker for diagnosis nor for monitoring disease progression of PD and MSA.

The uniqueness of our patient cohort is the major strength of our study. Only patients with diagnostic uncertainty at baseline were included in the study and their clinical diagnosis was re-evaluated after 3 and 12 years. Although this may also be regarded as a weakness, i.e., bias toward more complicated cases, this experimental setting exactly reflects the clinical situation when biomarkers are needed most, i.e., when there is diagnostic uncertainty during first visit.

Our study also had limitations. First, patients presented heterogeneity with respect to dopaminergic medication at the time of lumbar puncture. The majority of patients were tested drug-naïve or “off” medication. However, for some of the patients medication withdrawal was ethically not possible. Second, our group of MSA patients was relatively small, which may have affected our analyses; especially for the correlation between the biomarkers and disease progression parameters. Third, the final diagnosis was based on clinical evaluation according to international diagnostic criteria but has not been confirmed by postmortem neuropathologic examination. This may have caused potential misdiagnoses, but we have reduced this risk by the very long follow-up of the patients. Some concerns might arose regarding the long storage of the CSF samples previous to analysis (23 years). However, a study has shown the stability of long-term storage of CSF samples in Biobanks^14^, which reinforce the quality and reliability of our results.

In summary, our results indicate that MCP-1 levels in CSF cannot distinguish between PD, MSA, and controls, but correlate with disease progression in the PD group, suggesting its usefulness as part of a bigger panel to predict motor dysfunction in PD.

## Methods

### Patients

A total of 46 PD and 17 MSA patients were selected based on CSF availability from a prospective cohort study performed at the Radboud University Medical Center (Nijmegen, the Netherlands)^38^. In this study, 156 patients, referred to our center between January 2003 to December 2006 because of parkinsonism and diagnostic uncertainty, were included. Exclusion criteria were age younger than 18 years, history of brain surgery or neurodegenerative disease other than parkinsonism or unstable comorbidity. All patients underwent a structured interview, detailed and standardized neurologic examination, blood collection, lumbar puncture and other ancillary investigations (i.e., brain magnetic resonance imaging (MRI), ^123^I-iodobenzamide single photon-emission computed tomography, analysis of CSF, and anal sphincter electromyography) within 6 weeks after inclusion. The study design, methods and patient population have been extensively described elsewhere^38^. These patients were followed up for 3 years and a final clinical diagnosis was established by two expert neurologists in movement disorders based on a repeated structured interview and extensive neurological examination. In 2018, 12 years after inclusion, all diagnoses were re-evaluated and updated by two independent neurologists, according to the most recent clinical criteria^38–42^ and based on clinical data collected at time of inclusion and at 3- and 10-year follow-up. The diagnoses of some patients may have changed over time as illustrated in Supplementary Fig. 1. Disease severity and cognitive function were evaluated using the Hoehn and Yahr (HY) scores, the Unified Parkinson’s Disease Rating Scale part III (UPDRS-III), the International Cooperative Ataxia Rating Scale (ICARS) and the Mini-Mental State Examination (MMSE). Baseline clinical scores were assessed during “off” dopaminergic medication: before start or after 3 weeks of withdrawal of dopaminergic therapy. Clinical scores after 3 years of follow-up were assessed at least 12 h after the last oral dose of dopaminergic medication, unless skipping dopaminergic medication led to unacceptable deterioration of symptoms for the participant. Disease progression at 3-year follow-up was assessed by subtracting the score at follow-up visit after 3 years from the score at baseline and dividing by years of follow-up (3 years) (Table 1).

**Table 1:** Demographics.

	Controls	PD	MSA			*p* value^*ǂ*^
			MSA-P	MSA-C	MSA-P/C	
			14	2	1	
*N*	52	46	17			–
Age (years at inclusion)	64.4 ± 8.7	57.5 ± 10.0	61.6 ± 7.9			0.004
Sex (male/female)	24/28	30/16	12/5			0.11
Disease duration since first symptoms (months)	N.A.	35.5 ± 32.75	29.9 ± 24.4			0.77
L-DOPA medication at LP (no/past/yes/n.d.)	N.A.	31/7/6/2	9/4/2/2			–
Disease severity						
UPDRS-III score	N.A.	27.3 ± 12.7 (45)	30.2 ± 11.2 (17)			0.036
HY score	N.A.	2.0 ± 0.7 (45)	2.5 ± 1.0 (17)			0.007
ICARS score	N.A.	2.8 ± 3.2 (42)	9.5 ± 11.1 (13)			0.010
MMSE score	N.A.	28.3 ± 2.1 (46)	27.9 ± 2.5 (16)			0.60
Disease progression (3-year time-window)						
UPDRS-III score	N.A.	1.6 ± 4.6 (40)	0.7 ± 2.6 (7)			0.57
HY score	N.A.	0.1 ± 0.2 (43)	0.4 ± 0.3 (12)			0.005
ICARS score	N.A.	0.2 ± 1.2 (36)	1.9 ± 3.4 (9)			0.06
MMSE score	N.A.	−0.2 ± 0.8 (36)	−0.5 ± 0.5 (8)			0.05
Survival after 12 years (dead/alive)	N.A.	10/36	15/2			–

Data are represented as mean ± SD (*N*).

*MSA* multiple system atrophy, *PD* Parkinson’s disease, *MSA-P* multiple system atrophy parkinsonian type, *MSA-C* multiple system atrophy cerebellar type, *MSA-P/C* multiple system atrophy mixed parkinsonian and cerebellar, *N* number of patients per group, *N.A.* not applicable, *LP* lumbar puncture, *no* patients never took L-DOPA medication, *past* patients were off L-DOPA for 3 weeks before CSF collection, *yes* patients were on L-DOPA medication at CSF collection, *n.d*. not data, *HY* Hoehn and Yahr score, *UPDRS-III* Unified Parkinson’s Disease Rating Scale part III (motor score), *ICARS* International Cooperative Ataxia Rating Scale, *MMSE* Mini-Mental State Examination.

^*ǂ*^Chi-square test for sex differences, Kruskal–Wallis test for age and Mann–Whitney test with Bonferroni correction for the other parameters. *p* value was considered significant when <0.05.

The control group consisted of 52 patients aged above 40 years with neither a neurological nor an inflammatory disease and who underwent a lumbar puncture because of a suspected neurological disorder that was subsequently ruled out in the diagnostic process.

### Ethical approval

This study was approved by the Central Committee on Research Involving Human Subjects in the region Arnhem-Nijmegen (2002/188) and all participants provided written informed consent.

### Cerebrospinal fluid samples

Lumbar puncture was performed as described previously^38^. CSF samples had no blood contamination (leukocyte count fewer than 5 cells/μL and erythrocyte count fewer than 200 cells/μL)^43^. CSF was immediately frozen at −80 °C and only thawed once for aliquoting and analysis previously to measurements. CSF samples from either PD or MSA patients were obtained in the same period and were all treated similarly.

### ELISAs

MCP-1 and NLK levels in CSF were measured in 2019, i.e., 23 years after CSF withdrawal. MCP-1 levels in CSF were measured using a human MCP-1 ELISA Kit (ab100586, Abcam, Cambridge, UK). CSF samples were diluted eight times and measurements were performed according to company’s recommendations. We validated the reliability of the ELISA kit for CSF analysis by measuring the limit of detection (LOD = 1.28 pg/mL), the lower limit of quantification (LLOQ = 6.48 pg/mL) the dilutional linearity (108 ± 16%; range: 4–427 pg/mL), the intraplate variation (coefficient of variation (CV): 7.2 ± 5.2%; *n* = 3, range: 26.6–38.6 pg/mL), the inter-assay variation (CV: 10.9 ± 5.7%; *n* = 3), the intra-assay variation (CV: 6.0 ± 2.2%; *n* = 7) and the parallelism (100–120%)^44^.

NLK levels in CSF were measured using the human Glucose-6-Phosphate Isomerase ELISA Kit (ab171575, Abcam). CSF samples were diluted six times and measurements were performed according to the manufacturer’s recommendations. To validate the reliability of the kit for CSF analysis we measured the LOD (0.06 ng/mL), LLOQ (0.14 ng/mL), the dilutional linearity (109 ± 10%; range: 0.25–3.76 ng/mL), the intra-assay variation (CV: 3.6% ± 3.6; n = 102) and the inter-assay variation (CV: 4.9 ± 2.9%; *n* = 5).

All samples, for both MCP-1 and NLK, were measured in duplicate and the CV was calculated. Five CSF quality control (QC) samples were included in duplicate in all measurements to correct for interplate variation. Briefly, a correction factor was calculated per plate using QC concentrations by dividing the concentration of the QC on the reference plate (i.e., the plate with the lowest %CV between duplicates) by the same QC on the other plates. Then, the average of the five QC correction factors was calculated per plate and multiplied by the protein levels of the samples of that specific plate.

### Data analysis

Statistical analyzes were performed using IBM SPSS Statistics (v.25.0.0.1). Two-sided Kruskal–Wallis test or Mann–Whitney test with Bonferroni correction was performed to assess differences between groups for age, baseline and follow-up parameters, as well as disease progression. Chi-square test was used to assess sex differences. Group comparison of MCP-1 and NLK concentration in CSF was performed by rank analysis of covariance to correct for age. Briefly, the dependent variables and the covariate were ranked. Then, a linear regression of the ranks of the dependent variable on the ranks of the covariate was performed and the unstandardized residuals were saved. Finally, a two-sided ANOVA with Hochberg correction for multiple testing was performed using the unstandardized residuals. Disease progression was calculated using annual change in HY, UPDRS, ICARS, and MMSE scores using the 3-year follow-up and baseline scores. Spearman’s test was used to correlate the levels of biomarkers at baseline with the annual progression scores, as well as disease severity at baseline and at 3-year follow-up. In all cases, a *p* value < 0.05 was considered as statistically significant.

### Reporting summary

Further information on experimental design is available in the Nature Research Reporting Summary linked to this article.

## Supplementary information

Supplementary figure 1

Reporting Summry

## Data Availability

The datasets used and analyzed during the current study are available from the corresponding author on reasonable request.

## References

[1] Wirdefeldt K, Adami HO, Cole P, Trichopoulos D, Mandel J (2011). Epidemiology and etiology of Parkinson’s disease: a review of the evidence. Eur. J. Epidemiol..

[2] Ali K, Morris HR (2015). Parkinson’s disease: chameleons and mimics. Pract. Neurol..

[3] Dorsey ER, Bloem BR (2018). The Parkinson pandemic—a call to action. JAMA Neurol..

[4] Dorsey ER, Sherer T, Okun MS, Bloem BR (2018). The emerging evidence of the Parkinson pandemic. J. Parkinsons Dis..

[5] Kalia LV, Lang AE (2015). Parkinson’s disease. Lancet.

[6] Ross GW (2004). Parkinsonian signs and substantia nigra neuron density in decendents elders without PD. Ann. Neurol..

[7] Braak H (2003). Staging of brain pathology related to sporadic Parkinson’s disease. Neurobiol. Aging.

[8] Fanciulli A, Wenning GK (2015). Multiple-system atrophy. N. Engl. J. Med..

[9] Krismer F, Wenning GK (2017). Multiple system atrophy: insights into a rare and debilitating movement disorder. Nat. Rev. Neurol..

[10] Hoglinger GU (2017). Differentiation of atypical Parkinson syndromes. J. Neural Transm..

[11] Hoehn MM, Yahr MD (2001). Parkinsonism: onset, progression, and mortality. 1967. Neurology.

[12] Halliday G, Hely M, Reid W, Morris J (2008). The progression of pathology in longitudinally followed patients with Parkinson’s disease. Acta Neuropathol..

[13] Tufekci KU, Meuwissen R, Genc S, Genc K (2012). Inflammation in Parkinson’s disease. Adv. Protein Chem. Struct. Biol..

[14] Reale M (2009). Peripheral cytokines profile in Parkinson’s disease. Brain Behav. Immun..

[15] Sokolova A (2009). Monocyte chemoattractant protein-1 plays a dominant role in the chronic inflammation observed in Alzheimer’s disease. Brain Pathol..

[16] Melah KE (2016). Cerebrospinal fluid markers of Alzheimer’s disease pathology and microglial activation are associated with altered white matter microstructure in asymptomatic adults at risk for Alzheimer’s disease. J. Alzheimer’s Dis..

[17] Gurney M (1986). Neuroleukin: a lymphokine product of lectin-stimulated T cells. Science.

[18] Chaput M (1988). The neurotrophic factor neuroleukin is 90% homologous with phosphohexose isomerase. Nature.

[19] Iannicola C (2000). Early alterations in gene expression and cell morphology in a mouse model of Huntington’s disease. J. Neurochem..

[20] Tanie Y, Tanabe N, Kuboyama T, Tohda C (2018). Extracellular neuroleukin enhances neuroleukin secretion from astrocytes and promotes axonal growth in vitro and in vivo. Front. Pharmacol..

[21] Lee W-J (2018). Plasma MCP-1 and cognitive decline in patients with Alzheimer’s disease and mild cognitive impairment: a two-year follow-up study. Sci. Rep..

[22] Romagnoli A (2003). Neuroleukin inhibition sensitises neuronal cells to caspase-dependent apoptosis. Biochem. Biophys. Res. Commun..

[23] Sawyer AJ (2014). The effect of inflammatory cell-derived MCP-1 loss on neuronal survival during chronic neuroinflammation. Biomaterials.

[24] Liu W, Gao Y, Chang N (2017). Nurr1 overexpression exerts neuroprotective and anti-inflammatory roles via down-regulating CCL2 expression in both in vivo and in vitro Parkinson’s disease models. Biochem. Biophys. Res. Commun..

[25] Jabbari E (2019). Proximity extension assay testing reveals novel diagnostic biomarkers of atypical parkinsonian syndromes. J. Neurol. Neurosurg. Psychiatry.

[26] Lindqvist D (2013). Cerebrospinal fluid inflammatory markers in Parkinson’s disease–associations with depression, fatigue, and cognitive impairment. Brain Behav. Immun..

[27] Wennstrom M (2015). The inflammatory marker YKL-40 is elevated in cerebrospinal fluid from patients with Alzheimer’s but not Parkinson’s disease or dementia with lewy bodies. PLoS ONE.

[28] Rydbirk R (2017). Cytokine profiling in the prefrontal cortex of Parkinson’s disease and multiple system atrophy patients. Neurobiol. Dis..

[29] Santaella A (2020). Inflammation biomarker discovery in Parkinson’s disease and atypical parkinsonisms. BMC Neurol..

[30] Schroder JB (2018). Immune cell activation in the cerebrospinal fluid of patients with Parkinson’s disease. Front Neurol..

[31] Magdalinou NK (2015). A panel of nine cerebrospinal fluid biomarkers may identify patients with atypical Parkinsonian syndromes. J. Neurol. Neurosurg. Psychiatry.

[32] Chen X, Hu Y, Cao Z, Liu Q, Cheng Y (2018). Cerebrospinal fluid inflammatory cytokine aberrations in Alzheimer’s disease, Parkinson’s disease and amyotrophic lateral sclerosis: a systematic review and meta-analysis. Front. Immunol..

[33] Lee WJ (2018). Plasma MCP-1 and cognitive decline in patients with Alzheimer’s disease and mild cognitive impairment: a two-year follow-up study. Sci. Rep..

[34] Regnault A (2019). Does the MDS-UPDRS provide the precision to assess progression in early Parkinson’s disease? Learnings from the Parkinson’s progression marker initiative cohort. J. Neurol..

[35] Holden SK, Finseth T, Sillau SH, Berman BD (2018). Progression of MDS-UPDRS scores over five years in de novo Parkinson disease from the Parkinson’s progression markers initiative cohort. Mov. Disord. Clin. Pract..

[36] Skorvanek M (2017). Differences in MDS-UPDRS scores based on Hoehn and Yahr stage and disease duration. Mov. Disord. Clin. Pract..

[37] Knight AL (2014). The glycolytic enzyme, GPI, is a functionally conserved modifier of dopaminergic neurodegeneration in Parkinson’s models. Cell Metab..

[38] Aerts MB (2015). Ancillary investigations to diagnose parkinsonism: a prospective clinical study. J. Neurol..

[39] Postuma RB (2015). MDS clinical diagnostic criteria for Parkinson’s disease. Mov. Disord..

[40] Postuma RB (2016). The new definition and diagnostic criteria of Parkinson’s disease. Lancet Neurol..

[41] Hoglinger GU (2017). Clinical diagnosis of progressive supranuclear palsy: the movement disorder society criteria. Mov. Disord..

[42] Gilman S (2008). Second consensus statement on the diagnosis of multiple system atrophy. Neurology.

[43] Müller M, Kuiperij HB, Claassen JA, Küsters B, Verbeek MM (2014). MicroRNAs in Alzheimer’s disease: differential expression in hippocampus and cell-free cerebrospinal fluid. Neurobiol. Aging.

[44] Andreasson U (2015). A practical guide to immunoassay method validation. Front. Neurol..

