# Supplementary material for: Cerebrospinal fluid monocyte chemoattractant protein 1 correlates with progression of Parkinson’s disease
Source: NPJ Parkinsons Dis. 2020 Sep 3;6:21. doi: 10.1038/s41531-020-00124-z (PMC7471278; doi:10.1038/s41531-020-00124-z)
Supplement: Supplementary file 1 — Supplementary figure 1 [file 41531_2020_124_MOESM1_ESM.pdf]

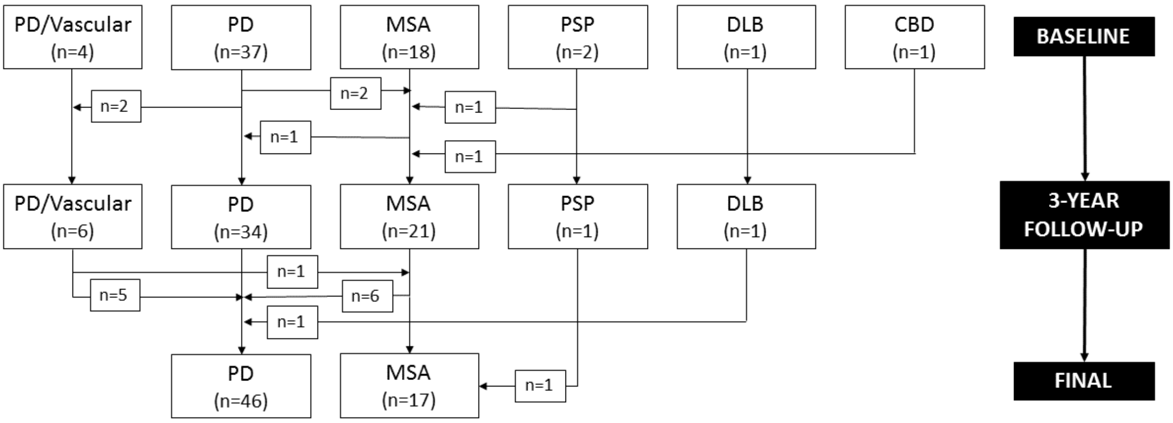

Supplementary figure 1. Change of diagnosis during follow-up. *Arrows reflect the number of patient for whom the diagnosis changed. PD/Vascular: Parkinson's diseases with vascular parkinsonism; PD: Parkinson's disease; MSA: multiple system atrophy; PSP: Progressive Supranuclear Palsy; DLB: Dementia with Lewy Bodies; CBD: corticobasal degeneration; n: number of patients.*
