# Supplementary material for: Cerebrospinal fluid monocyte chemoattractant protein 1 correlates with progression of Parkinson’s disease
Source: NPJ Parkinsons Dis. 2020 Sep 3;6:21. doi: 10.1038/s41531-020-00124-z (PMC7471278; doi:10.1038/s41531-020-00124-z)
Supplement: Supplementary file 2 — Reporting Summry [file 41531_2020_124_MOESM2_ESM.pdf]

nature research

Corresponding author(s): DBRP NPJPARKD-00385

Last updated by author(s): Apr 7, 2020

Reporting Summary

Nature Research wishes to improve the reproducibility of the work that we publish. This form provides structure for consistency and transparency in reporting. For further information on Nature Research policies, see Authors & Referees and the Editorial Policy Checklist.

Statistics

For all statistical analyses, confirm that the following items are present in the figure legend, table legend, main text, or Methods section.

n/a	Confirmed	
☐	☒	The exact sample size (*n*) for each experimental group/condition, given as a discrete number and unit of measurement
☐	☒	A statement on whether measurements were taken from distinct samples or whether the same sample was measured repeatedly
☐	☒	The statistical test(s) used AND whether they are one- or two-sided<br>*Only common tests should be described solely by name; describe more complex techniques in the Methods section.*
☐	☒	A description of all covariates tested
☐	☒	A description of any assumptions or corrections, such as tests of normality and adjustment for multiple comparisons
☐	☒	A full description of the statistical parameters including central tendency (e.g. means) or other basic estimates (e.g. regression coefficient) AND variation (e.g. standard deviation) or associated estimates of uncertainty (e.g. confidence intervals)
☐	☒	For null hypothesis testing, the test statistic (e.g. *F*, *t*, *r*) with confidence intervals, effect sizes, degrees of freedom and *P* value noted<br>*Give P values as exact values whenever suitable.*
☒	☐	For Bayesian analysis, information on the choice of priors and Markov chain Monte Carlo settings
☒	☐	For hierarchical and complex designs, identification of the appropriate level for tests and full reporting of outcomes
☒	☐	Estimates of effect sizes (e.g. Cohen's *d*, Pearson's *r*), indicating how they were calculated

Our web collection on statistics for biologists contains articles on many of the points above.

Software and code

Policy information about availability of computer code

Data collection	No software was used
Data analysis	IBM SPSS Statistics (v.25.0.0.1)

For manuscripts utilizing custom algorithms or software that are central to the research but not yet described in published literature, software must be made available to editors/reviewers. We strongly encourage code deposition in a community repository (e.g. GitHub). See the Nature Research guidelines for submitting code & software for further information.

Data

Policy information about availability of data

All manuscripts must include a data availability statement. This statement should provide the following information, where applicable:

- Accession codes, unique identifiers, or web links for publicly available datasets
- A list of figures that have associated raw data
- A description of any restrictions on data availability

The datasets used and analyzed during the current study are available from the corresponding author on reasonable request indefinitely after publication date.

Field-specific reporting

Please select the one below that is the best fit for your research. If you are not sure, read the appropriate sections before making your selection.

☒ Life sciences ☐ Behavioural & social sciences ☐ Ecological, evolutionary & environmental sciences

For a reference copy of the document with all sections, see nature.com/documents/nr-reporting-summary-flat.pdf

October 2018

Life sciences study design

All studies must disclose on these points even when the disclosure is negative.

Sample size	No sample size-calculation was performed. We used all PD and MSA patients from which we had enough cerebrospinal fluid available.
Data exclusions	No data were excluded.
Replication	All ELISA measurements were performed in duplicate.<br>Diagnoses were established by two independent neurologist.
Randomization	Cerebrospinal fluid of the different patients groups was randomly allocated in the ELISA plates.
Blinding	The investigators were blinded to the diagnosis of the patients during ELISA data collection.

Reporting for specific materials, systems and methods

We require information from authors about some types of materials, experimental systems and methods used in many studies. Here, indicate whether each material, system or method listed is relevant to your study. If you are not sure if a list item applies to your research, read the appropriate section before selecting a response.

Materials & experimental systems

n/a	Involved in the study	
☐	☒	Antibodies
☒	☐	Eukaryotic cell lines
☒	☐	Palaeontology
☒	☐	Animals and other organisms
☐	☒	Human research participants
☒	☐	Clinical data

Methods

n/a	Involved in the study	
☒	☐	ChIP-seq
☒	☐	Flow cytometry
☒	☐	MRI-based neuroimaging

Antibodies

Antibodies used	MCP-1 ELISA Kit (ab100586, Abcam, Cambridge, UK) and human Glucose-6-Phosphate Isomerase ELISA Kit (ab171575, Abcam)
Validation	We validated the reliability of the kits for cerebrospinal fluid analysis by measuring the LOD, LLOQ, dilutional linearity, intra-assay variation and inter-assay variation.

Human research participants

Policy information about studies involving human research participants

Population characteristics	A total of 46 PD, 17 MSA patients and 52 controls were selected for the study. The sex ratio between groups was similar. Age was different between groups and we corrected for the covariate age in our statistical analysis.
Recruitment	Patients referred to our center (Radboudumc, Nijmegen, The Netherlands) between January 2003 to December 2006 because of parkinsonism and diagnostic uncertainty
Ethics oversight	This study was approved by the Central Committee on Research Involving Human Subjects in the region Arnhem-Nijmegen (2002/188) and all participants provided written informed consent.

Note that full information on the approval of the study protocol must also be provided in the manuscript.
